# Why we should use simpler models if the data allow this: relevance for ANOVA designs in experimental biology

**DOI:** 10.1186/1472-6793-8-16

**Published:** 2008-07-21

**Authors:** Stanley E Lazic

**Affiliations:** 1 Department of Physiology, Development and Neuroscience, University of Cambridge, CB2 3DY, UK; 2 Centre for Brain Repair, University of Cambridge, CB2 2PY, UK

## Abstract

**Background:**

Analysis of variance (ANOVA) is a common statistical technique in physiological research, and often one or more of the independent/predictor variables such as dose, time, or age, can be treated as a continuous, rather than a categorical variable during analysis – even if subjects were randomly assigned to treatment groups. While this is not common, there are a number of advantages of such an approach, including greater statistical power due to increased precision, a simpler and more informative interpretation of the results, greater parsimony, and transformation of the predictor variable is possible.

**Results:**

An example is given from an experiment where rats were randomly assigned to receive either 0, 60, 180, or 240 mg/L of fluoxetine in their drinking water, with performance on the forced swim test as the outcome measure. Dose was treated as either a categorical or continuous variable during analysis, with the latter analysis leading to a more powerful test (p = 0.021 vs. p = 0.159). This will be true in general, and the reasons for this are discussed.

**Conclusion:**

There are many advantages to treating variables as continuous numeric variables if the data allow this, and this should be employed more often in experimental biology. Failure to use the optimal analysis runs the risk of missing significant effects or relationships.

## Background

Analysis of variance (ANOVA) is a commonly used statistical technique in experimental biology. Often one or more of the independent/predictor variables such as dose, time, or age, can be treated as a continuous numeric variable rather than a categorical variable during analysis, even if experimentally it is treated as a category. For example, animals may be randomly assigned to one of several different groups, each of which receives a different dose of a drug (including a control group which receives no drug). This would commonly be analysed with a one-way ANOVA, with one control group and several experimental groups. Dose would be treated as a categorical variable when testing whether the drug had any effect on the response variable, such as performance on a behavioural test. Another example is killing animals at different ages in order to assess how age affects anatomical or physiological variables of interest. Animals could be killed at perhaps three different ages (young, middle, and old), and again this would be traditionally analysed with a one-way ANOVA. Alternatively, dose or age could be treated as a continuous variable and these analyses would proceed as a simple regression analysis, with both the response and predictor variables being numeric. Pharmacologists and toxicologists routinely treat dose as a numeric variable and fit nonlinear dose-response curves to the data, but apart from these specific disciplines, this method of analysis is not common in experimental biology (but would be the method of choice for a statistician). There are a number of advantages of such an approach when used appropriately, such as greater statistical power due to more precise estimates, a simpler and more informative interpretation of the results, a more parsimonious explanation of the data with fewer parameters, and transformations of the predictor variable are possible. To simplify the discussion, the first type of analysis will be referred to as the *ANOVA *analysis and the second as the *regression *analysis (which is understood to be a *linear *regression unless otherwise indicated), as most readers will be familiar with these terms. However, the only difference between them is whether the predictor variable is treated as a categorical factor or a continuous numeric variable, and both are specific cases of a linear model [1].

This paper will discuss the advantages of using a regression analysis instead of the more common ANOVA analysis, why these advantages occur, and when this analysis is, and is not, appropriate. In addition, an example is provided illustrating how the incorrect conclusion can be reached using the standard ANOVA analysis.

## Results and Discussion

### Increased power when treating dose as a continuous variable

Twenty rats were randomly assigned to four groups and given either 0, 60, 180, or 240 mg/L of fluoxetine in their drinking water. After four weeks, performance on the forced swim test (FST) was assessed, and the amount of time spent immobile was the main response variable of interest (data are presented in Table 1. The data were analysed twice, once treating dose as a categorical factor (ANOVA analysis) and once as a continuous numeric variable (regression analysis).

**Table 1:** Raw immobility scores (seconds) for twenty rats at different doses of fluoxetine.

Dose	0	80	160	240
	182	158	140	163
	112	165	135	183
	206	168	110	25
	170	182	128	100
	164	97	155	61

Mean	166.8	154.0	133.6	106.4

**Figure 1:**
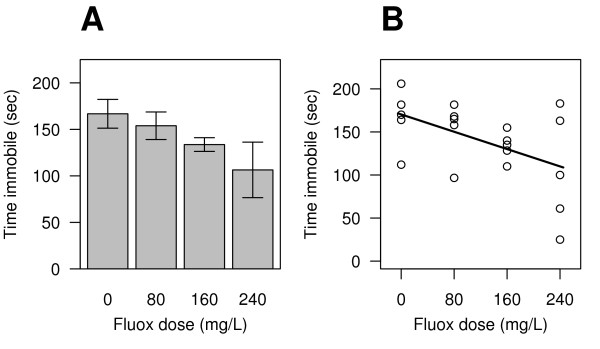
Effect of fluoxetine on the forced swim test. Data of this type are typically plotted as means ± the standard error of the mean and analysed with an one-way ANOVA (A). The data are also plotted as individual points with a regression line (B). In both cases, it is evident that the time spent immobile decreases with increasing fluoxetine.

It is evident that there is a dose-dependent relationship between fluoxetine and immobility time (Fig 1), with decreased immobility associated with higher doses of fluoxetine. A standard method of analysing this data is with a one-way ANOVA, with one control and three treatment groups. This analysis leads to the conclusion that there is no significant effect of fluoxetine (p = 0.157; Table 2). However, when dose is treated as a continuous variable, the effect of fluoxetine becomes significant (p = 0.020). To understand why this occurred, it is necessary to understand how the two analyses are implemented. The difference between them is that the ANOVA analysis estimates four parameters from the data (one mean, and 3 differences between means), while the regression analysis only estimates two parameters (one intercept, and one slope). Since one degree of freedom (*df*) is lost every time a parameter is estimated, the ANOVA analysis has lost two more dfs compared to the regression analysis. The ANOVA tables of both analyses are presented in Table 2. The sum of squares (*SS*) for dose is slightly greater with the ANOVA than the regression method (10,420 versus 10,161), indicating that the ANOVA analysis accounted for slightly more variation in immobility times. This is also reflected in the residual *SS*, with the regression analysis having a slightly larger *SS*_*Residual*_, indicating a greater amount of unexplained variation. It would appear therefore that the ANOVA analysis is preferable because it accounts for slightly more of the variation. However, the ANOVA method is evaluated on 3 and 16 *df*, while the regression method is evaluated on 1 and 18 *df*, and this makes all the difference. The mean square (*MS*) for dose is calculated as *MS*_*Dose *_= *SS*_*Dose*_/*df*_*Dose *_and having 3 *df *in the ANOVA analysis reduces *SS*_*Dose *_by a third, whereas in the regression analysis *MS*_*Dose *_= *SS*_*Dose *_(dividing by one *df*). The opposite occurs with the residuals: because *SS*_*Residual *_is divided by a bigger number in the regression analysis (*df *= 18) than the ANOVA analysis (*df *= 16), *MS*_*Residual *_will be smaller with the regression analysis. This is important because the F-value is calculated as *F *= *MS*_*Dose*_/*MS*_*Residual*_, and therefore the higher *MS*_*Dose *_and the lower *MS*_*Residual*_, the higher the F-value. As can be seen, the F-value is 6.46/1.98 = 3.26 times bigger using the regression analysis. The critical value of F (F-crit) is the number that the calculated F-value has to exceed in order to be significant at the 0.05 level, and it is different for each method because it is based on the degrees of freedom. F-crit for the regression analysis is 4.41/3.24 = 1.36 times bigger, but is still less than the change in F-value, and explains why the regression method is more powerful. This will be true in general, and more formally, the linear regression model

**Table 2:** ANOVA tables for the two analyses.

		df	SS	MS	F value	F-crit	P value
		
ANOVA	Dose	3	10 420	3 473	1.98	3.24	0.157
	Residuals	16	28 043	1 753			
	Total	19	38 463				
Regression	Dose	1	10 161	10 161	6.46	4.41	0.020
	Residuals	18	28 303	1 572			
	Total	19	38 464				

The difference in the last digit of Total SS between the ANOVA and regression analysis is due to rounding error.

(1) *y*_*i *_= *α *+ *βx*_*i *_+ *ε*_*i*_

where *α *is the *y*-intercept and *β *the slope of the regression line, is a special case of the ordinary 'factor' model

(2) *y*_*ij *_= *μ *+ *θi *+ *ε*_*ij*_

where *μ *is the grand mean and *θ*_*i *_is the treatment effect of the *i *th group (the difference between the grand mean and the mean of each group). In each case, the residuals (*ε*) are normally distributed with a mean of zero. We would always choose (1) in preference to (2) if the data allow this (see below), as this allows the parameters to be estimated more accurately (see reference [2] for a formal proof).

The FST is a standard behavioural screen for antidepressant drugs [3,4], and the effects of fluoxetine on this test are well documented [5,6]. Kulkarni and Dhir estimated 18 mg/kg of fluoxetine as the dose that produces a response in 50% of rodents (ED_50_), when given intraperitoneally [6]. This corresponds to the 160 mg/L group in the present experiment. The lack of statistical significance therefore reflects the reduced power of the ANOVA analysis compared to the regression analysis; using the ANOVA analysis, we would falsely retain the null hypothesis that fluoxetine has no effect on this behavioural test (Type II error).

Readers might wonder if the regression method will increase the chance of false-positives (Type I errors), since it can have greater power and therefore provides lower p-values than the ANOVA method. The answer is no, because the Type I error rate is set by the researcher before looking at the data (traditionally *α *= 0.05). The preference for the regression method (when appropriate) is in line with the 'classical' Neyman-Pearson method of analysis commonly used in experimental biology: set the probability of falsely rejecting the null hypothesis when it is true (*α *= 0.05), and then use the test with the highest probability of rejecting the null hypothesis when the alternative hypothesis is true (i.e. the test with the highest power) [7]. The aim is to minimise Type II errors (failing to detect a significant difference when one exists), subject to the constraint on Type I errors [8]. The problem with the ANOVA analysis is that it has reduced power to detect linear relationships (and hence larger p-values), which can lead to increased Type II errors.

### A simpler and more informative interpretation

The regression analysis also lends itself to a simpler interpretation. The effect of fluoxetine on immobility time on the FST can be stated thus: for every 100 mg/L increase in fluoxetine, immobility time decreases by 25 seconds (CI_95% _= 4 to 46 seconds; p = 0.021). This is true for doses between zero and 240 mg/L, and extrapolating to higher doses should be done with caution. This states the relationship between the two variables, and the confidence interval provides an estimate of uncertainty. Contrast this with interpreting the ANOVA analysis; one can only describe qualitatively that there was a decrease in immobility time with higher doses of fluoxetine (and provide the p-value), but this is much less informative than quantifying this relationship as in the regression analysis. The amount of variation in the response variable accounted for by the predictor variable could also be mentioned (R^2 ^= 0.271), but this is still not as informative. *Post hoc *tests are routinely applied in such situations, whether the overall ANOVA was significant or not. These are problematic (in this case and arguably many others) because they reflect lack of power rather than a lack of effect, particularly if corrections for multiple comparisons are used. For example, the only significant difference was between the control and 240 mg/L group when uncorrected pairwise comparisons are used (p = 0.037). This is no longer significant when correcting for multiple tests.  Comparing the control group to all the fluoxetine groups gives p = 0.090 for this comparison (Dunnett's test) or blindly doing all pairwise comparisons gives p = 0.221 (Bonferroni correction). Each pairwise comparison uses half the total sample size (comparing two groups of n = 5) and correcting for multiple comparisons raises the bar for significance, making the power of such tests greatly reduced. All of this can be avoided with the regression analysis; not only is the interpretation simpler, but more informative.

Another drawback of the ANOVA analysis is that the results are invariant to the ordering of the groups. For example, the above ANOVA result would be identical if the data from the 80 and 240 mg/L groups were swapped, such that the 80 mg/L group now has the lowest immobility time. There is now no consistent decrease in immobility time with increasing dose (i.e. no dose-response) and therefore less evidence for an effect of fluoxetine, yet the p-value remains the same (p = 0.157). This is because '0', '80', '160' and '240' are just labels in the ANOVA analysis, which could just as easily have been the labels A to D. The regression analysis has the advantage of respecting the order of the data, where zero means no drug, and 160 mg/L is twice 80 mg/L.

### Greater parsimony

Other things being equal, it is generally accepted that a simpler explanation is preferable to a more complex one [9]. The regression analysis describes the data with just two parameters: the slope and intercept of the regression line. In contrast, the ANOVA analysis requires four parameters: the mean of the control group (0 mg/L), and the difference between the control and each of the fluoxetine groups (using treatment contrasts). The ANOVA analysis therefore uses twice as many parameters to describe the data, and therefore other things being equal, the regression analysis is preferred because it is simpler.

### Transformations of the predictor are possible

Since the predictor in the regression analysis is treated as a number and not a category, it is possible to transform it. For example, a toxicology study may use doses that span several orders of magnitude (e.g. 0.01, 0.1, 1, 10, 100 mg/kg), but it is not predicted that the response will have such a wide range (e.g. the proportion of animals surviving is bounded by zero and one), and therefore it is unlikely that the relationship between the two will be linear. However, taking the log_10 _of the above doses gives values of -2, -1, 1, 2, and 3, which are more likely to be related linearly to the response variable. Treating the predictor as a continuous variable therefore provides added flexibility by allowing transformations that the ANOVA analysis does not.

### When not to use the regression analysis

The first requirement is that the predictor variable must in fact be continuous, and a true categorical variable such as different types of drug, arbitrarily labelled from 1 to 4, cannot be treated as a continuous variable. Second, the regression analysis requires the relationship between the response and predictor variables to be linear. Nonlinearity could be handled by transforming one or more of the variables (see above), but it may be preferable to use the ANOVA analysis in this case if it makes the interpretation simpler. Alternatively, the relationship between the response and predictor might be 'U'- or inverted 'U'-shaped, in which case the ANOVA analysis would be preferable (of course, a quadratic term could be added to the regression analysis or a nonlinear regression could be used, but these will not be considered further here). The greater the extent of nonlinearity, the less power (and greater lack of fit) the regression analysis will have compared to the ANOVA analysis, and the optimal fit for the regression line would occur when it passes through the mean of each group.

The relationship between the response and predictor variables can be established by plotting the data as in Figure 1B. However, plotting the fitted values against the residuals will also provide information on the lack of fit of the regression model. Crawley [[10], p. 415–417] suggests that the lack of fit of the regression model can be tested by using both the continuous and categorical variables in the same analysis; entering the continuous variable first, and then the categorical factor. If p < 0.05 for the categorical factor, then the ANOVA model is preferred to the regression model. Note that this is a sequential (Type I SS), where the continuous variable is entered into the model first, and the categorical variable accounts for any *additional *variation not accounted for by the continuous variable. For some statistical software, the default SS will have to be changed to 'Type I' before the analysis is carried out. It is also possible to compare the two models directly with an F-ratio using the following equation

(3) $$F=\frac{\left(SS_{reg}-SS_{ANOVA})/(df_{reg}-df_{ANOVA}\right)}{SS_{ANOVA}/df_{ANOVA}}$$

where *SS*_*reg *_and *SS*_*ANOVA *_are the residual sums of squares from the regression and ANOVA analyses, and *df*_*reg *_and *df*_*ANOVA *_are the residual degrees of freedom. This follows an F-distribution with *df*_*reg *_- *df*_*ANOVA *_degrees of freedom in the numerator and *df*_*ANOVA *_degrees of freedom in the denominator. With the present data, the results are F_2,16 _= 0.074, p = 0.929, indicating that the ANOVA model does not provide a significantly better fit than the regression model.

In addition, the two models can be compared using Akaike's information criterion (AIC) [11], and a discussion of this approach can be found in Motulsky and Christopoulos [12] (which can also be obtained at ). Briefly, AIC is a measure of how well a model fits the data. The more parameters a model has, the better the fit; however, AIC penalises superfluous parameters and thus represents a trade-off between goodness-of-fit and the number of parameters. The model with the lowest AIC value is preferred, and AIC for the ANOVA method was 211.7, while for the regression method it was 207.9, indicating that the regression method is preferred. A related approach is the Bayesian information criterion (BIC, [13]), which tends to penalise complex models more heavily than the AIC, and therefore gives greater preference to models with fewer parameters [14]. The BIC value is interpreted in a similar manner to the AIC (the model with the lower value is preferred), and BIC for the ANOVA method was 216.7, while for the regression method it was 210.8. Therefore, using the F-ratio, AIC, and BIC, it is possible to compare both the ANOVA and regression models directly to see which is preferable.

The final requirement is that the groups must be independent, with different animals (or subjects, samples, etc.) in each group (i.e. dose is a 'between-subjects' factor). Neither the one-way ANOVA nor the regression analysis are appropriate if the same subjects give values at more than one level of the factor; for example, if a response was measured at more than one time point. In this case, a repeated-measures ANOVA or a mixed-effects model [15,16] should be used.

### Extensions and further applications

The example provided had only a single predictor variable, but the results and the general approach also apply to higher order designs where one or more variables could be treated as continuous rather than categorical, leading to analysis of covariance (ANCOVA) or multiple regression-type analyses. For example, if the present data contained both males and females, separate regression lines could be fit for each sex, each with its own slope and intercept. It is important to note that if there is no significant interaction between the continuous variable (dose) and the groups (sex), then the interaction term should be removed from the model, which constrains the regression lines to have equal slopes. If this is not done, the interpretation of differences between sexes becomes difficult, and can lead to erroneous conclusions [17,18]. In addition, the response variable may be counts, proportions, percentages, or other types of data that would normally be analysed with a generalised linear model, and the advantages of treating the predictor variable as continuous rather than categorical are similar for these analyses [19]. A related issue is that of 'data carving' [20], where a continuous numeric (often non-experimental) variable such as age is binned into a few categories; for example, age may be dichotomised into young and old groups based on a median-split, or perhaps a middle group would be included as well, and the data would then be analysed with an ANOVA. There is little to be gained from such an approach and it is not recommended [20].

In general, the greater the number of groups, the greater the usefulness of using the regression analysis; for example, if there were eight groups *SS*_*Dose *_would be divided by 8 - 1 = 7 *df *for the ANOVA analysis, but *MS*_*Dose *_would still equal *SS*_*Dose *_(dividing by one *df*) for the regression analysis. At the other extreme, the two analyses would always produce identical results if there are only two groups (an independent samples T-test would normally be used in such a case, and would also give identical results).

The analysis of datasets such as the one presented in this paper are not limited to ANOVA and regression analyses, computationally intensive methods (e.g. permutation tests, bootstrapping, etc.), non-parametric tests (e.g. Kruskal-Wallis test) and tests for trend (e.g. Pitman test) are also available, and each have their associated advantages, disadvantages, and caveats. The advantages of the regression analysis are well known to statisticians, but relatively unknown to experimental laboratory-based biologists. This is likely due to regression and ANOVA being treated as separate topics in undergraduate statistics courses aimed at natural scientists (and the reason why these terms were used to describe the different analyses in this paper). In addition, many modern point-and-click statistics packages maintain this distinction, with the tests located under different sub-menus. However, regression and ANOVA are not two fundamentally different analyses; they both fit (different) linear models to the data, and it is up to the analyst to decide which model is preferable in each case.

## Conclusion

As demonstrated above, treating an experimental variable as continuous rather than categorical during analysis has a number of advantages. First, it will generally have greater statistical power. Second, because fewer parameters are used to describe the data, it is more parsimonious. Third, it often provides a simpler interpretation (e.g. a change in the predictor variable by *x *units leads to a change in the response variable of *y *units), and this is usually more informative as well. Finally, there is the added flexibility of allowing transformations of the predictor variable. Because of these advantages, treating independent variables as continuous should be the method of choice in the first instance, with ANOVA being used if regression analysis is not appropriate (e.g. if the relationship between the variables is not in fact linear).

Failing to use the optimal analysis runs the risk of missing significant effects; in the example provided, the ANOVA analysis did not reject the null hypothesis while the regression analysis did. It is not known how many published studies failed to find significant effects, or how many studies have not been published due to lack of significant results (file-drawer problem) because an ANOVA analysis was used when a regression analysis would have been more powerful. But given the ubiquity of ANOVA in experimental biology, it is likely a non-trivial number. It is hoped that, when feasible, readers will employ this approach in their own research to improve the power of their analyses and arrive at a better understanding of their data.

## Methods

### Animals

Twenty male Sprague-Dawley rats (age = 8 weeks) were obtained from Harlan Ltd., UK. Upon arrival at the animal facility, rats were individually housed and allowed to acclimatise for one week. Rats were then randomly divided into four groups (n = 5 per group) and given 0, 80, 160, or 240 mg/L of fluoxetine in their drinking water, along with saccharine. This corresponds to approximate doses of 0, 10, 18, and 25 mg/kg of body weight [21]. Ambient temperature was maintained at 21°C and humidity at 55%. Rats had *ad libitum *access to food and water and were kept on a reversed 12-hour light/dark cycle (lights off at 10:00 AM). After four weeks of fluoxetine treatment, behavioural testing on the forced swim test was conducted and rats were killed the following day. Animal work conformed to the UK Animals (Scientific Procedures) Act 1986 and was performed under appropriate Home Offce project and personal licenses.

### Forced swim test

Rats were placed into a clear perspex swim tank (height = 40 cm, inner diameter = 19 cm) filled with warm water to 20 cm for 5 min. The latency to immobility and time spent immobile were measured. Immobility time is taken to be a measure of behavioural 'despair', and antidepressants typically decrease the amount of time rodents spend immobile.

### Statistical analysis

Analysis was conducted with R (version 2.6.0) [22,23]. The relationship between fluoxetine and performance on the FST was analysed with a linear model (using the 'lm' function with the default treatment contrasts). A p-value of less than 0.05 was considered statistically significant. Data are provided in Table 1 and R code is available so that readers may reproduce the analysis [see Additional file 1]. Note that the results have been rounded to sensible values for presentation in the text and in Table 2.

## Abbreviations

ANCOVA: analysis of covariance; ANOVA: analysis of variance; df: degrees of freedom; ED_50_: effective dose 50%; FST: forced swim test; MS: mean square; SS: sum of squares

## Supplementary Material

Additional file 1 **Code for analysis**. R code is provided as a plain text file. Click here for file
